# Three-dimensional nonlinear model of rock creep under freeze–thaw cycles

**DOI:** 10.1371/journal.pone.0287605

**Published:** 2023-07-06

**Authors:** Yanting Wang, Dong Wang, Guanghe Li, Laigui Wang, Chun Zhu, Yongzhi Du, Zhiwei Zhou

**Affiliations:** 1 College of Mining, Liaoning Technical University, Fuxin, China; 2 School of Mechanics and Engineering, Liaoning Technical University, Fuxin, China; 3 School of Earth Sciences and Engineering, Hohai University, Nanjing, China; 4 Guoneng Baorixile Energy Co., Ltd., Hulun Buir, China; University of Science and Technology Beijing, CHINA

## Abstract

In areas with large differences between day and night temperature, the freeze–thaw cycle and frost heaving force in rock mass generate cracks within the rock, which seriously threatens the stability and safety of geotechnical engineering structures and surrounding buildings. This problem can be solved by developing a reasonable model that accurately represents the rock creep behavior. In this study, we developed a nonlinear viscoelastic–plastic creep damage model by introducing material parameters and a damage factor while connecting an elastomer, a viscosity elastomer, a Kelvin element, and a viscoelastic–plastic element in series. One- and three-dimensional creep equations were derived, and triaxial creep data were used to determine the model parameters and to validate the model. The results showed that the nonlinear viscoelastic–plastic creep damage model can accurately describe rock deformation in three creep stages under freeze–thaw cycles. In addition, the model can describe the time-dependent strain in the third stage. Parameters *G*_1_, *G*_2_, and *η*_20_’ decrease exponentially with the increase in the number of freeze–thaw cycles while parameter *λ* increases exponentially. These results provide a theoretical basis for studying the deformation behavior and long-term stability of geotechnical engineering structures in areas with large diurnal temperature differences.

## 1 Introduction

In the past few years, the number and scale of open-pit mines in China have increased rapidly. The number of open-pit mines in Inner Mongolia, Xinjiang, Gansu, and other regions in Northwest China was 83.39% of the national count in 2021. Rock mass engineering in these areas is faced with complex geographical and climatic conditions such as seasonally frozen soil and low temperatures [1]. The large variations in temperature due to seasonal alternations and the day–night cycle result in varying degrees of freeze–thaw disasters such as frost heave cracking, freeze–thaw collapse, and slope instability [2, 3]. These disasters directly affect rock engineering stability and safety and pose serious challenges to engineering construction and subsequent maintenance [4–6]. Therefore, in-depth studies on the aging mechanical behavior and creep law of rock specimens under freeze–thaw environments are of vital theoretical significance for enriching the creep mechanics theory and analyzing the time-dependent strain of rocks.

Previous research has mostly focused on the physical and mechanical behavior and creep deformation law of rocks under freeze–thaw cycles [7–9], and important results have been obtained through experimental analyses, quantitative evaluations, and engineering practice. According to the Lemaitre strain equivalence hypothesis, Yuan et al. [10] set the initial damage condition of intact rock as the reference damage condition to construct a damage constitutive model. Yan et al. [11] separated the strain of rock under the action of freeze–thaw cycles into initial damage strain, additional damage strain, and plastic strain, and the authors established a damage constitutive model of the elastic–plastic freeze–thaw cycle. Considering the theory of continuous damage mechanics, Zhang et al. [12] constructed a damage model of freeze–thaw loading, factoring in changes in confining pressure. In terms of the Lemaitre strain equivalence hypothesis, Chen et al. [13] established a jointed rock mass composite damage model, considering macroscopic and microscopic defects. Liu et al. [14] used the definition of damage and the Lemaitre strain equivalent equation to establish a freeze–thaw damage evolution equation in the constant creep stage. According to the von Mises yield criterion, a damage formula for freeze–thaw and creep changes throughout the nonlinear creep stage was constructed. The Burgers model parameters were corrected by introducing damage variables to construct the creep damage model under the action of freeze–thaw cycles. Li [15] constructed a rock freeze–thaw damage element based on nuclear magnetic resonance porosity and a creep damage element based on volumetric strain; both elements were introduced into classical rheological elements to construct a nonlinear creep damage model for describing the freeze–thaw behavior of rocks. Zhang et al. [16] conducted shear creep experiments and mesoscopic characteristics analyses, proposed rock freeze–thaw damage viscous components, and established a granite freeze–thaw shear creep constitutive model for characterizing the influence of freeze–thaw cycles on shear creep behavior. Through a triaxial creep test, Wan et al. [17] established a Nishihara model based on damage mechanics principles and extended the model to a three-dimensional form according to the elastic–plastic mechanics theory. Song et al. [18] performed triaxial creep experiments on saturated red sandstone under freeze–thaw cycles and established a freeze–thaw damage creep model considering both freeze–thaw cycles and damage effects. Hou et al. [19] combined statistical damage mechanics theory and the maximum tensile strain failure criterion to establish a freeze–thaw rock damage model. Zhang et al. [20] performed mesoscopic characteristics analyses and shear creep tests on granite specimens undergoing different freeze–thaw cycles. They found that the increase in the number of freeze–thaw cycles increased the damage degree of the rock surface, and a creep model of freeze–thaw coupling damage and stress damage was constructed to describe the rock creep characteristics.

Evidently, key results have been obtained in the study of the mechanical characteristics and creep deformation behavior of rocks under freeze–thaw cycles. In this study, we constructed a nonlinear viscoelastic–plastic creep damage model. Material parameters and the damage factor were introduced into the model, as well as an elastomer, a viscosity elastomer, a Kelvin element, and a viscoelastic-plastic element connected in series. The one- and three-dimensional creep equations were derived, and triaxial creep data were used to obtain the model parameters and validate the model. The findings of this study can provide a scientific basis and technical reference for the support maintenance and long-term stability analysis of geotechnical engineering structures in Northwest China, which has seasonally frozen soil and an extremely low-temperature complex climate environment.

## 2 Analysis of triaxial creep test of mudstone under freeze–thaw cycles

Li [21] conducted triaxial creep experiments of mudstone specimens under 0, 10, 20, and 30 freeze–thaw cycles. In this study, we put all the mudstone specimens into the vacuum saturation equipment for 48 h under negative pressure saturation before conducting the freeze–thaw test. The maximum and minimum temperature limits were 20°C and -20°C, respectively. Chen’s loading method was used for the triaxial rock creep test, and an LVDT displacement meter was used for displacement monitoring. The confining pressure was maintained at 5 MPa, and the axial loading was divided into five levels: 10 MPa, 20 MPa, 30 MPa, 40 MPa, and 50 MPa. The loading rate was controlled by the force control mode to 30 kN/min approximately 0.25 MPa/s), and the load was applied to each creep stability stage for 18 h.

**Fig 1:**
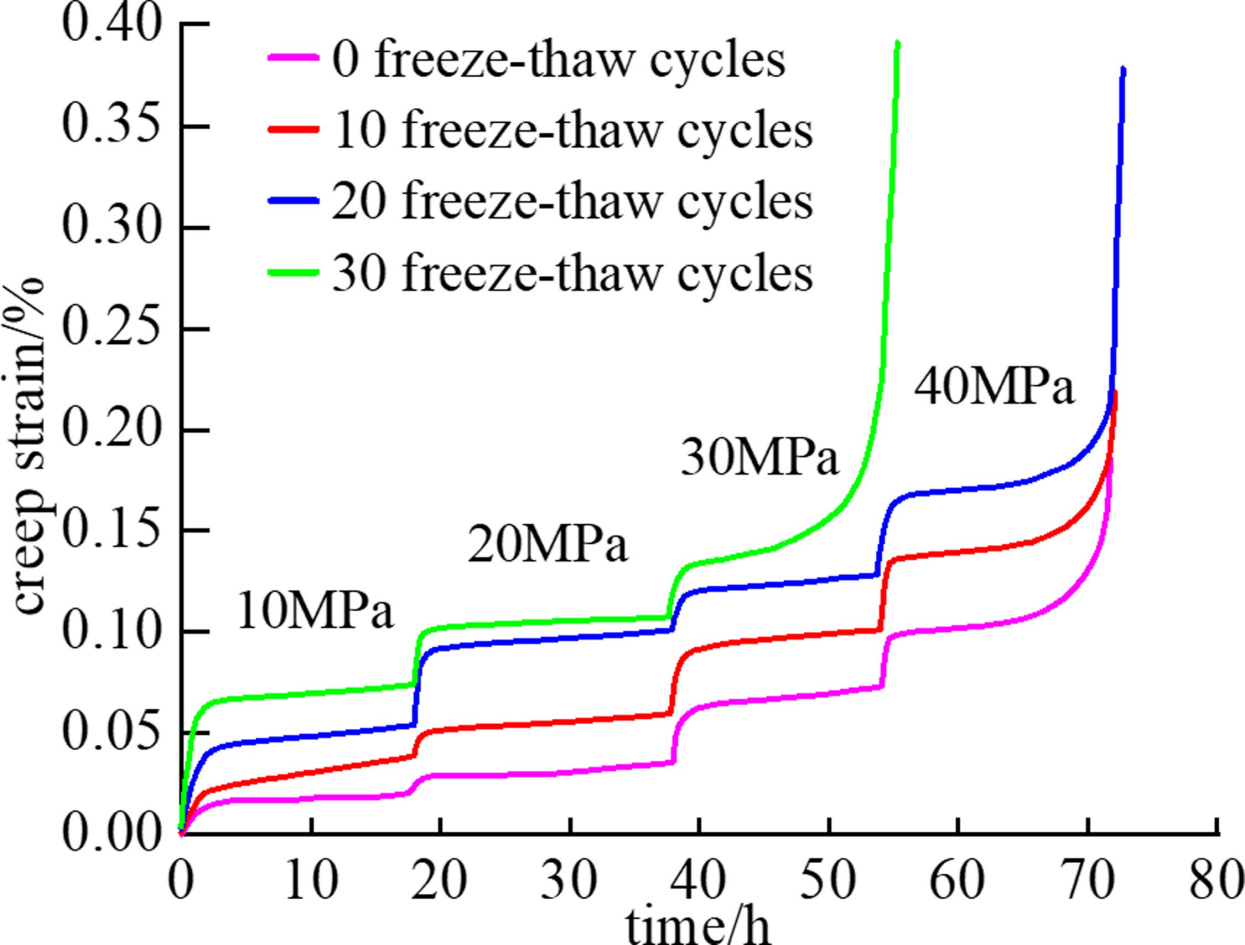


Fig 1 shows the creep curves of mudstone under different freeze–thaw cycles, and the curves exhibit the typical creep deformation behavior [22] of three creep stages: attenuation creep stage, constant creep stage, and accelerated creep stage. Under each loading stage, the rock specimens underwent elastic deformation or viscoelastic deformation due to the sudden increase in axial load, which is approximately expressed as a vertical or high slope line segment on the creep curve. The first deformation stage is characterized by a slow increase in mudstone deformation and a decrease in creep deformation. Thus prior to the second stage, the mudstone deformation increases gradually while the creep rate tends to be stable. The first and second stages are characterized by a few internal mudstone cracks and the growth and germination of micro cracks. In these stages, the mudstone damage is minimal, and no obvious damage crack appears on the mudstone surface. When the stress on the rock exceeds the yield strength of the rock, the rock specimen enters the third deformation stage. In this stage, the axial deformation and deformation rate of the mudstone increase rapidly, and macroscopic cracks appear until failure.

## 3 Establishment of nonlinear viscoelastic–plastic creep damage model and derivation of creep equation

**Fig 2:**
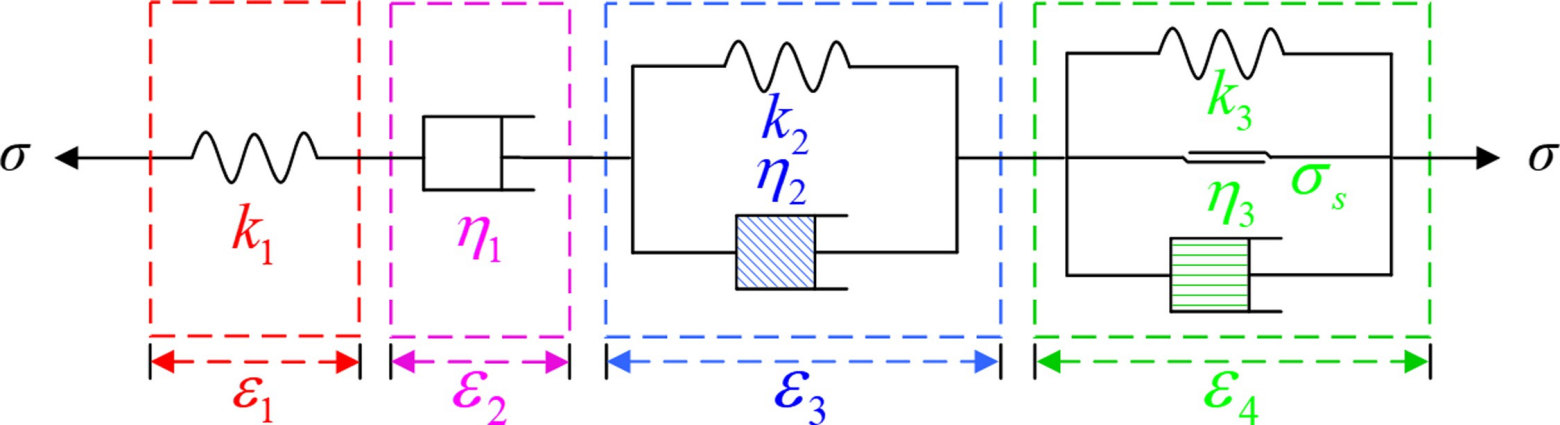


In the first and second stages, zero or negligible damage occurs in the rock specimen; in the third stage, damage gradually accumulates in the rock and breaks the rock, showing obvious nonlinear characteristics [23, 24]. The classical Burgers model and the Nishihara model, which are composed of ideal elements such as an elastomer, a Kelvin element, and a viscosity element, can only model the rock creep behavior rather than the entire creep process. Thus, describing the creep properties of the third stage is a key scientific problem [25]. Therefore, according to existing results of the Burgers model, we modeled the creep behavior in the third stage by considering the time correlation of the viscosity coefficient of the Kelvin element; we also used the viscoelastic–plastic element considering damage (Fig 2).

### 3.1 One-dimensional creep equation

#### 3.1.1 Burgers model

The classical Burgers creep model is a typical four-element model, which comprises an elastomer, a viscosity element, and a Kelvin element connected in series. According to the series rule, the total strain of the Burgers model can be expressed as:

$$\varepsilon^{\prime }(t)=\varepsilon_{1}(t)+\varepsilon_{2}(t)+\varepsilon_{3}(t),$$ (1)

where *ε*’(*t*) is the strain of the Burgers model, %; *ε*_1_(*t*) is the strain of the elastomer, %; *ε*_2_(*t*) is the strain of the viscosity element, %; and *ε*_3_(*t*) is the strain of the Kelvin element, %.

By summing the strains of each element, the Burgers model [26] creep equation can be expressed as:

$$\varepsilon^{\prime }(t)=\frac{\sigma }{k_{1}}+\frac{\sigma }{\eta_{1}}t+\frac{\sigma }{k_{2}}\left(1-\exp(-\frac{k_{2}}{\eta_{2}}t)\right),$$ (2)

where *σ* is the stress, MPa; *k*_1_ is the elastic modulus of the elastomer, MPa; *η*_1_ is the viscosity coefficient of the viscosity element, MPa·h; *k*_2_ is the elastic modulus of the Kelvin element, MPa; and *η*_2_ is the viscosity coefficient of the Kelvin element, MPa·h.

In the first stage, with the increase in time, the unit creep of the rock gradually decreases while the creep curve slope gradually decreases. The entire stage exhibits nonlinear characteristics. In the second stage, the attenuation, characteristics, and time are closely related to the rock stress. During the creep test, the bonding structures in the rock are gradually damaged under constant stress; macroscopically, the creep strain increases gradually. The viscosity parameters increase with time in the first stage but gradually tend to be stable in the second stage, indicating that the rock creep reaches a stable state in the second stage [27, 28]. Although the traditional Kelvin element can better depict the creep attenuation law under a given stress, it fails to accurately reflect the nonlinear creep behavior under different stresses because the viscosity parameter remains constant with time. The correlation between the viscosity coefficient and time in the Kelvin element were considered in this study. It is assumed that the coefficient and time in the creep process are power functions [29]. The differential constitutive equation of the nonlinear Kelvin element is

$$\left\{\begin{array}{c} \sigma =k_{2}\varepsilon_{3}(t)+\eta_{2}(t){\dot{\varepsilon }}_{3}(t)\\ \eta_{2}(t)=\eta_{20}t^{1-\lambda } \end{array}\right.,$$ (3)

where *η*_20_ is the initial viscosity coefficient of the nonlinear Kelvin element, MPa·h; *η*_2_(*t*) is the viscosity coefficient of the nonlinear Kelvin element, MPa·h^2^; and *λ* is a constant.

Differentiating Eq (3) yields the creep equation (Eq (4)) of the nonlinear Kelvin element:

$$\varepsilon_{3}(t)=\frac{\sigma }{k_{2}}\left[1-\exp(-\frac{k_{2}}{\eta_{\text{20}}\lambda }t^{\lambda })\right].$$ (4)

#### 3.1.2 Viscoelastic–plastic element considering damage

When the loading level exceeds the long-term length, damage occurs in the rock and gradually develops over time [30]. Compared with the initial and constant creep stages, the accelerated creep stage accounts for a small part of the creep test process. The accelerated creep stage has a short duration but produces significant nonlinear deformation [31]. Therefore, to accurately describe the creep behavior of the third creep stage, we introduce the damage variable to the viscosity coefficient and the elastic modulus to the viscoelastic element to model the rock damage evolution mechanism [32]:

$$\left\{\begin{array}{c} D\text{=}\frac{\text{1-}\eta_{\text{30}}(D,t)}{\eta_{\text{30}}}\\ D\text{=}\frac{\text{1-}k_{\text{30}}(D,t)}{k_{\text{30}}} \end{array}\right..$$ (5)

Then,

$$\left\{\begin{array}{c} \eta_{\text{30}}(D,t)=\eta_{\text{30}}(1-D)\\ k_{\text{30}}(D,t)=k_{\text{30}}(1-D) \end{array}\right.,$$ (6)

where *D* is the damage factor; *η*_30_ is the initial viscosity coefficient of the viscoelastic–plastic element considering damage, MPa·h; *η*_30_(*D*,*t*) is the viscosity coefficient with damage variable, MPa·h; *k*_30_ is the initial elastic modulus of the viscoelastic–plastic element considering damage, MPa; and *k*_30_(*D*,*t*) is the elastic modulus with damage variable, MPa.

For simplicity, the classical creep damage function [33] is improved using the simplified damage expression commonly used internationally [34–36], and the damage factor *D* is given as Eq (7) [37, 38]:

$$D=1-e^{-\alpha t},$$ (7)

where *e* is the natural constant, and *α* is the material constant.

We can obtain the constitutive equation of this element considering damage as Eq (8) [39]:

$$\sigma =k_{30}(1-D)\varepsilon_{3}+\eta_{30}(1-D){\dot{\varepsilon }}_{3}+\sigma_{s}.$$ (8)

From the integral solution of Eq (8), we can obtain the one-dimensional creep equation of the viscoelastic–plastic element considering damage:

$$\varepsilon_{\text{4}}\text{=}\frac{\sigma \text{-}\sigma_{s}}{k_{30}(1-D)}\left[1-\exp\left(-\frac{k_{30}}{\eta_{30}}t\right)\right].$$ (9)

Therefore, the one-dimensional creep equation of the nonlinear viscoelastic–plastic creep damage model is:

$$\varepsilon (t)=\left\{\begin{array}{cc} \frac{\sigma }{k_{1}}+\frac{\sigma }{\eta_{1}}t+\frac{\sigma }{k_{2}}\left[1-\exp(-\frac{k_{2}}{\eta_{\text{20}}\lambda }t^{\lambda })\right] & \sigma <\sigma_{s}\\ \frac{\sigma }{k_{1}}+\frac{\sigma }{\eta_{1}}t+\frac{\sigma }{k_{2}}\left[1-\exp(-\frac{k_{2}}{\eta_{\text{20}}\lambda }t^{\lambda })\right]+\frac{\sigma \text{-}\sigma_{s}}{k_{30}(1-D)}\left[1-\exp\left(-\frac{k_{30}}{\eta_{30}}t\right)\right] & \sigma \geq \sigma_{s} \end{array}\right..$$ (10)

### 3.2 Three-dimensional creep equation

In engineering practice, rock mass is typically in three dimensions with a complex stress state. Hence, the one-dimensional creep equation should be extended to the three-dimensional space to establish a three-dimensional creep equation, providing scientific support for accurately describing rock creep behavior [37]. Geotechnical engineering research has produced several key findings through element models. However, certain problems exist in constructing the three-dimensional rock creep equation and determining parameters of the test curve. These problems are as follows: (1) when the test curve of creep deformation under three-dimensional stress conditions is fitted with a one-dimensional constitutive equation, the model parameters have different physical meanings in one-dimensional and three-dimensional cases, and the parameters obtained are inaccurate because a one-dimensional constitutive equation does not consider the effect of confining pressure on rock creep characteristics. (2) The second problem is the incorrect use of model parameters. For example, the elastic or viscoelastic modulus of elasticity in a one-dimensional constitutive relation is different from the corresponding elastic or viscoelastic shear modulus in the three-dimensional constitutive equation. Thus, the elastic modulus *k* in the one-dimensional constitutive relation should be replaced by the elastic shear modulus *G* and bulk modulus *K* determined by *k* and *μ* in the three-dimensional constitutive relation [40]. (3) When establishing the three-dimensional creep equation, the stress under uniaxial compression is directly replaced by the deviatoric stress *S*_*ij*_ under triaxial compression. The relationship between the deviatoric stress *S*_*ij*_ and the long-term strength of rock under three-dimensional stress is used as the basis for judging whether the rock enters the third stage [41]. The rationality of this criterion is also questionable. To avoid these problems, a reasonable three-dimensional creep equation is derived below.

In a three-dimensional stress state, assume that the total strain of the nonlinear viscoelastic–plastic creep damage model is *ε*_*ij*_, the strain of the elastomer is $\varepsilon_{ij}^{1}$, the strain of the viscous element is $\varepsilon_{ij}^{2}$, the strain of the nonlinear Kelvin element is $\varepsilon_{ij}^{3}$, and the strain of the viscoelastic–plastic element considering damage is $\varepsilon_{ij}^{4}$. As all four elements are in series, the total strain is given as [42]:

$$\varepsilon_{ij}=\varepsilon_{ij}^{1}+\varepsilon_{ij}^{2}+\varepsilon_{ij}^{3}+\varepsilon_{ij}^{4}.$$ (11)

The stress *σ*_*ij*_ at any point in the rock in an elastic state is composed of spherical stress tensor *σ*_*m*_*δ*_*ij*_ and deviatoric stress tensor *S*_*ij*_. Similarly, the strain at any point is composed of spherical strain tensor *ε*_*ij*_ and deviatoric strain tensor *e*_*ij*_; hence, we have the following equation [43, 44]:

$$\left\{\begin{array}{c} \sigma_{ij}=S_{ij}+\delta_{ij}\sigma_{m}\\ \varepsilon_{ij}=e_{ij}+\delta_{ij}\varepsilon_{m} \end{array}\right.,$$ (12)

where *δ*_*ij*_ is the Kronecker tensor.

In accordance with the generalized Hooke’s law,

$$\left\{\begin{array}{c} \sigma_{m}=3K\varepsilon_{m}\\ S_{ij}=2G_{1}e_{ij} \end{array}\right.,$$ (13)

where *K* is the bulk modulus of the elastomer, MPa; and *G*_1_ is the shear modulus of the elastomer, MPa.

Therefore, the three-dimensional constitutive equation of the elastomer is [34]:

$$\varepsilon_{ij}^{1}=\frac{1}{2G_{1}}S_{ij}+\frac{1}{3K}\delta_{ij}\sigma_{m}.$$ (14)

The three-dimensional creep equation of the viscous element and the nonlinear Kelvin element can be obtained by imitating the creep equation under the one-dimensional stress state:

$$\left\{\begin{array}{c} \varepsilon_{ij}^{2}=\frac{1}{2\eta_{1}{}^{\prime }}S_{ij}t\\ \varepsilon_{ij}^{3}=\frac{1}{2G_{2}}\left[1-\exp(-\frac{G_{2}}{\eta_{\text{20}}{}^{\prime }\lambda }t^{\lambda })\right]S_{ij} \end{array}\right.,$$ (15)

where *η*_1_’ is the viscous shear coefficient of the viscous element, MPa·h; *G*_2_ is the shear modulus of the nonlinear Kelvin element, Mpa; and *η*_20_’ is the viscous shear coefficient of the nonlinear Kelvin element, MPa·h.

The three-dimensional creep relation of the viscoelastic–plastic element considering damage is given by:

$$\left\{\begin{array}{c} \varepsilon_{ij}^{4}=\frac{1}{2\eta_{30}^{\prime }(1-D)}\left(\phi \left(\frac{F}{F_{0}}\right)\right)\frac{\partial Q}{\partial \sigma_{ij}}\left[1-\exp\left(-\frac{G_{3}(1-D)}{\eta_{30}^{\prime }(1-D)}t\right)\right]=\frac{1}{2\eta_{30}^{\prime }e^{-\alpha t}}\left(\phi \left(\frac{F}{F_{0}}\right)\right)\frac{\partial Q}{\partial \sigma_{ij}}\left[1-\exp\left(-\frac{G_{3}}{\eta_{30}^{\prime }}t\right)\right]\\ \left(\phi \left(\frac{F}{F_{0}}\right)\right)=\left\{\begin{array}{cc} 0 & F<0\\ \varphi \left(\frac{F}{F_{0}}\right) & F\geq 0 \end{array}\right. \end{array}\right.,$$ (16)

Where *η*_30_’ is the viscous shear coefficient of the viscoelastic–plastic element considering damage, MPa·h; *F* is the rock yield function; *F*_0_ is the initial value of the yield function (to simplify the calculation, *F*_0_ = 1 [45]); *ɸ*(·) is a power function, which typically takes the power exponent *n* = 1 [46]; *Q* is the plastic potential function, which follows the associated mobility guidelines, namely *F* = *Q*; and *G*_3_ is the shear modulus of the viscoelastic–plastic element considering damage, MPa.

The deviatoric stress tensor has a large influence on rock yield during rock creep, whereas the spherical stress tensor has little effect [47, 48]. Therefore, the yield relation can be expressed in terms of the Mises function:

$$F=\sqrt{J_{2}}-\sigma_{s}/\sqrt{3},$$ (17)

where √J_2_ is the second invariant of the stress deviator.

In the conventional triaxial compression creep experiment (*σ*_1_≥*σ*_2_ = *σ*_3_), the following conditions are satisfied [49–51]:

$$\left\{\begin{array}{c} \sigma_{m}=\frac{1}{3}\left(\sigma_{1}+2\sigma_{3}\right)\\ S_{11}=\sigma_{1}-\sigma_{m}=\frac{2}{3}(\sigma_{1}-\sigma_{3})\\ \sqrt{J_{2}}=\frac{\sigma_{1}-\sigma_{3}}{\sqrt{3}} \end{array}\right..$$ (18)

Therefore, based on Eqs. (16), (17), and (18), the creep equation of the viscoelastic–plastic element considering damage under a three-dimensional stress state is:

$$\varepsilon_{ij}^{4}=\left\{\begin{array}{cc} 0 & \sigma <\sigma_{s}(F<0)\\ \frac{\sigma_{1}-\sigma_{3}-\sigma_{s}}{3\eta_{30}{}^{\prime }e^{-\alpha t}}\left[1-\exp\left(-\frac{G_{3}}{\eta_{30}{}^{\prime }}t\right)\right] & \sigma \geq \sigma_{s}(F\geq 0) \end{array}\right..$$ (19)

From Eqs. (11), (14), (15), and (19), the creep equation of the nonlinear viscoelastic–plastic creep damage model under a three-dimensional stress condition can be obtained:

$$\varepsilon_{ij}=\left\{\begin{array}{cc} \frac{\sigma_{1}-\sigma_{3}}{3G_{1}}+\frac{\sigma_{1}+2\sigma_{3}}{9K}+\frac{\sigma_{1}-\sigma_{3}}{3\eta_{1}{}^{\prime }}t\text{+}\frac{\sigma_{1}-\sigma_{3}}{3G_{2}}\left[1-\exp(-\frac{G_{2}}{\eta_{\text{20}}{}^{\prime }\lambda }t^{\lambda })\right] & \sigma <\sigma_{s}\\ \frac{\sigma_{1}-\sigma_{3}}{3G_{1}}+\frac{\sigma_{1}+2\sigma_{3}}{9K}+\frac{\sigma_{1}-\sigma_{3}}{3\eta_{1}{}^{\prime }}t\text{+}\frac{\sigma_{1}-\sigma_{3}}{3G_{2}}\left[1-\exp(-\frac{G_{2}}{\eta_{\text{20}}{}^{\prime }\lambda }t^{\lambda })\right]\text{+}\frac{\sigma_{1}-\sigma_{3}-\sigma_{s}}{3\eta_{30}{}^{\prime }e^{-\alpha t}}\left[1-\exp\left(-\frac{G_{3}}{\eta_{30}{}^{\prime }}t\right)\right] & \sigma \geq \sigma_{s} \end{array}\right..$$ (20)

## 4. Model validation and parameter identification

**Fig 3:**
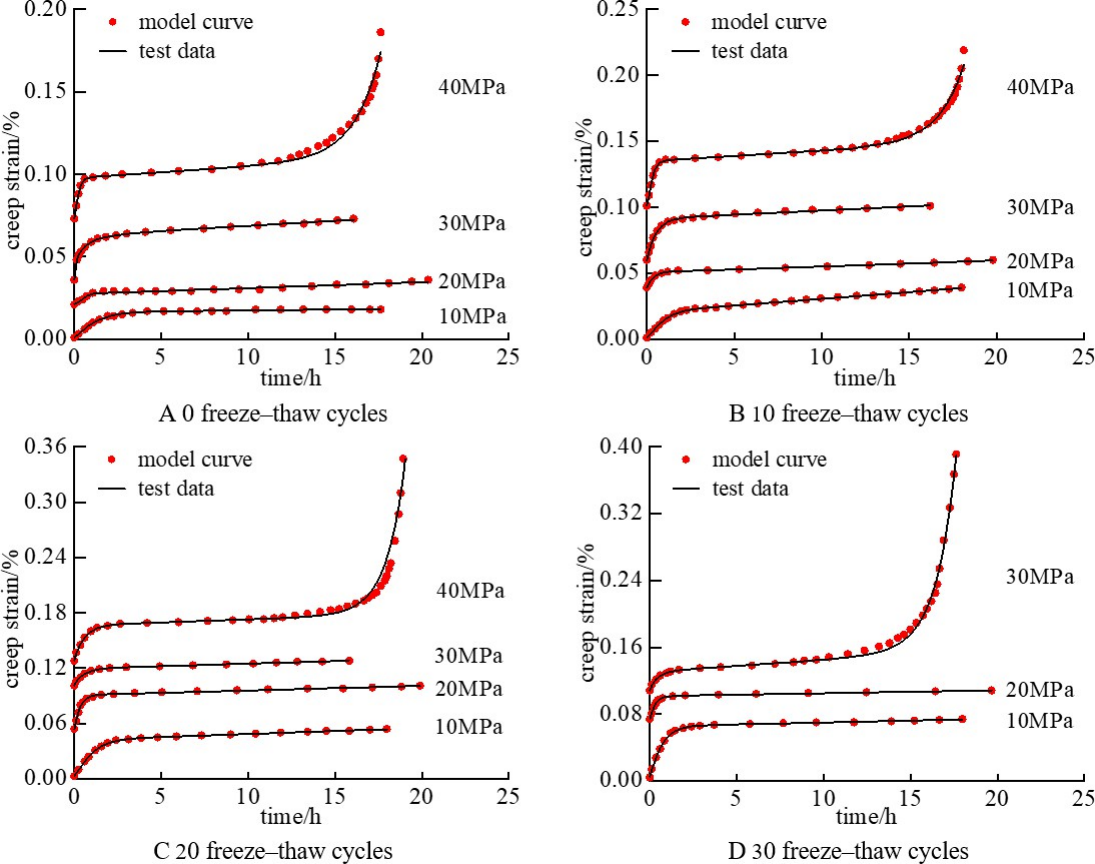


The rationality and accuracy of the derived model were verified through a rock creep test under freeze–thaw cycles, and the model parameters were determined using mudstone triaxial creep test data. To facilitate the data fitting, the model creep equation was simplified to Eqs. (21) and (22). The fitting results are illustrated in **Fig 3**, and the nonlinear viscoelastic–plastic creep damage model parameters are stated in Table 1.


$$A=\frac{\sigma_{1}-\sigma_{3}}{3G_{1}}+\frac{\sigma_{1}+2\sigma_{3}}{9K},B=\frac{\sigma_{1}-\sigma_{3}}{3},C=\frac{G_{2}}{\eta_{20}^{\prime }\lambda },E=\frac{\sigma_{1}-\sigma_{3}-\sigma_{s}}{3},H=\frac{G_{3}}{\eta_{30}{}^{\prime }}$$ (21)

$$\varepsilon_{ij}=\left\{\begin{array}{cc} A+\frac{B}{\eta_{1}^{\prime }}t+\frac{B}{G_{2}}\left[1-\exp\left(-Ct^{\lambda }\right)\right] & \sigma <\sigma_{s}\\ A+\frac{B}{\eta_{1}^{\prime }}t+\frac{B}{G_{2}}\left[1-\exp\left(-Ct^{\lambda }\right)\right]+\frac{E}{\eta_{30}^{\prime }e^{-\alpha t}}[1-\exp(-Ht)] & \sigma \geq \sigma_{s} \end{array}\right.$$ (22)

**Table 1:** Nonlinear viscoelastic–plastic creep damage model parameters.

Freeze–thaw cycles	Axial stress /MPa	*G*_1_/MPa	*η*_1_’/MPa·h	*G*_2_/MPa	*η*_20_’/MPa·h	*λ*	*η*_30_’/MPa·h	*G*_3_*/*MPa	*α*	*R* ^2^
0	10	5628.3954	16343.0370	107.8038	124.0330	1.1387				0.9985
	20	610.7000	24206.0265	523.4446	366.6561	0.8605				0.9846
	30	519.9437	27274.4123	592.3947	602.0958	0.6145				0.9995
	40	347.6532	16752.1662	471.9826	61.8639	1.2929	2.2858 × 10^5^	2.9958× 10^6^	0.6747	0.9823
10	10	3716.0670	1659.2195	87.5605	70.7054	1.1583				0.9994
	20	314.6641	11095.5321	241.7821	81.2849	0.9474				0.9991
	30	316.3328	13886.9997	294.9097	181.0846	0.7770				0.9987
	40	23.0512	13941.4723	348.2997	52.2850	1.4012	6.5505× 10^6^	3.0102× 10^7^	0.7127	0.9858
20	10	1603.2727	2610.9466	42.9805	39.0327	1.1684				0.9997
	20	228.1540	10302.7425	133.8073	48.6042	0.9551				0.9986
	30	209.0145	13775.9812	279.8865	165.5109	0.8370				0.9992
	40	197.5097	17892.9463	308.1372	179.7492	1.0002	7.0388× 10^8^	1.4292× 10^9^	0.9465	0.9802
30	10	1032.2214	3622.2356	27.9349	15.8775	1.1744				0.9995
	20	165.4859	14641.2629	180.3514	58.5334	0.9642				0.9989
	30	175.0463	15907.9129	341.1197	173.2693	0.8643	6.9194× 10^7^	1.6178× 10^10^	0.8750	0.9974

**Fig 4:**
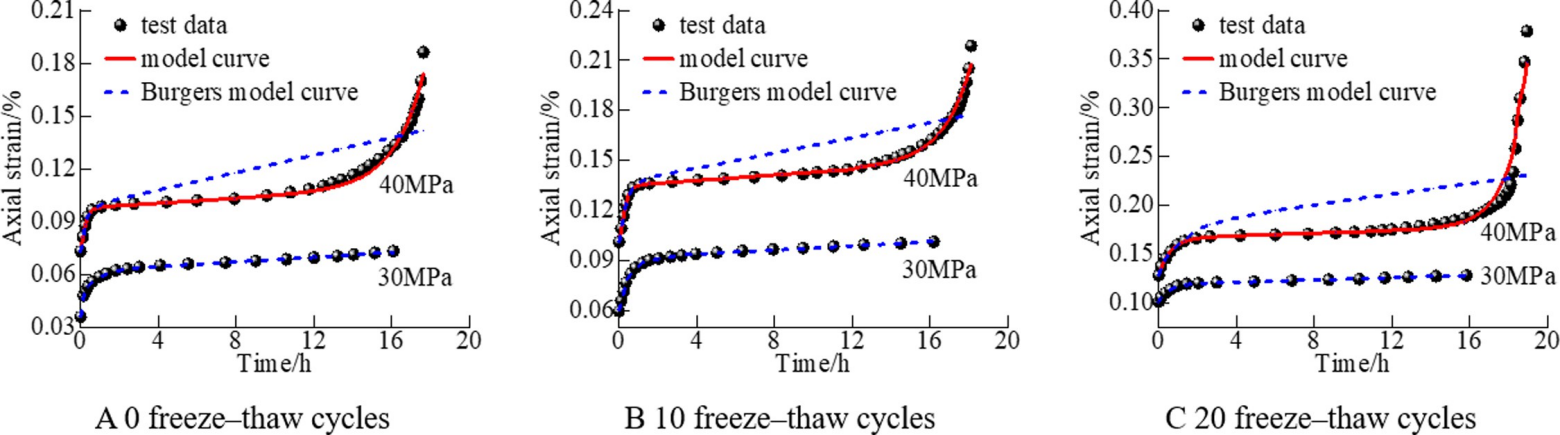


**Fig 3** illustrates a comparison between the mudstone test data and model curve under freeze–thaw cycles. The test data have a good fit with the nonlinear viscoelastic–plastic creep damage model curve, with a correlation coefficient close to 1, and the fitting results of the third stage are mostly consistent with the test data. This result shows that the model better represents rock mechanical properties in three stages under freeze–thaw cycles, and the model accurately describes creep characteristics. **Fig 4** illustrates a comparison between the derived model and the Burgers model fitted with the test data. The Burgers model accurately describes the data only in the attenuation and constant creep stages (30 MPa) but unsatisfactorily characterizes the test data in the acceleration stage (40 MPa). The derived model reflects the rock creep characteristics in all stages, especially in the accelerated creep stage. Moreover, the experimental data and fitting curve are in good agreement, and the fitting curve is smooth. This comparison further verifies the accuracy and applicability of the derived model.

The mechanism of rock freeze–thaw damage can be explained as follows. During the freeze–thaw cycle, the freezing of the water inside the rock expands the pores, and the volume expansion generates permanent cracks in the rock as the water melts. Consequently, numerous pores are created inside the rock, damaging the rock and weakening its mechanical properties. Therefore, with the gradual increase in the axial load and the increase in the number of freeze–thaw cycles, the nonlinear deformation characteristics of rock become more obvious while macroscopic cracks are generated faster until damage occurs.

**Fig 5:**
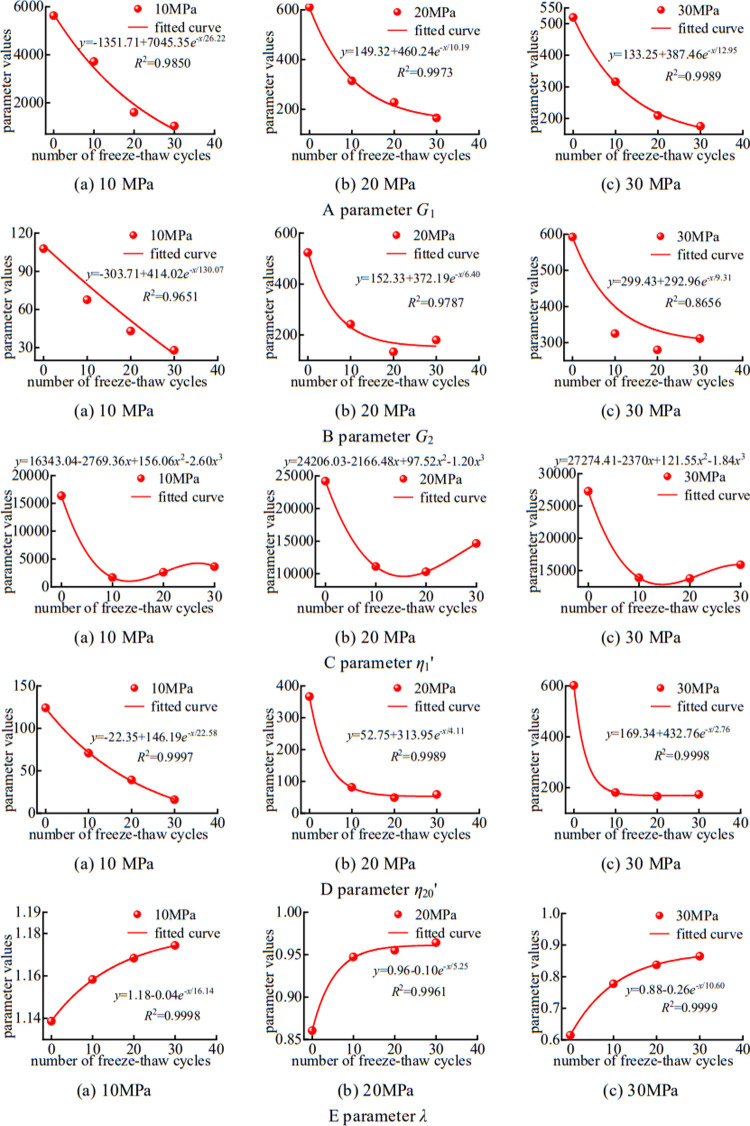


**Fig 5** illustrates the quantitative relationship between freeze–thaw times under axial loads and parameters *G*_1_, *G*_2_, *η*_1_’, *η*_20_’, and *λ*. The analysis shows that with the increase in the number of freeze–thaw cycles, *G*_1_, *G*_2_, and *η*_20_’ decrease exponentially whereas *λ* increases exponentially. Hence, the freeze–thaw cycle considerably influences creep deformation, and the influence gradually tends to be stable as the number of freeze–thaw cycles increases. The effect of freeze–thaw times on *η*_1_’ can be expressed by a cubic polynomial: *y* = *ax*^3^ + *bx*^2^ + *cx* + *d*. Table 2 shows the fitting function of each parameter with changes in the number of freeze–thaw cycles.

**Table 2:** Fitting function of parameters varying with freezing and thawing times.

Parameters	10 MPa	20 MPa	30 MPa
*G*_1_	*y* = -1351.71+7045.35*e*^-*x*/26.22^	*y* = 149.32+460.24*e*^-*x*/10.19^	*y* = 133.25+387.35*e*^-*x*/12.95^
	*R*^2^ = 0.9850	*R*^2^ = 0.9973	*R*^2^ = 0.9989
*G*_2_	*y* = -303.71+414.02*e*^-*x*/130.07^	*y* = 152.33+372.19*e*^-*x*/6.40^	*y* = 299.43+292.96*e*^-*x*/9.31^
	*R*^2^ = 0.9651	*R*^2^ = 0.9787	*R*^2^ = 0.8656
*η*_1_’	*y* = 16343.04–2769.36*x*	*y* = 24206.03–2166.48*x*	*y* = 27274.41-2370*x*
	+156.06*x*^2^-2.60*x*^3^	+97.52*x*^2^-1.20*x*^3^	+121.55*x*^2^-1.84*x*^3^
*η*_20_’	*y* = -22.35+146.19*e*^-*x*/22.58^	*y* = 52.75+313.95*e*^-*x*/4.11^	*y* = 169.34+432.076*e*^-*x*/2.76^
	*R*^2^ = 0.9997	*R*^2^ = 0.9989	*R*^2^ = 0.9998
*λ*	*y* = 1.18–0.04*e*^-*x*/16.14^	*y* = 0.96+0.10*e*^-*x*/5.25^	*y* = 0.88–0.26*e*^-*x*/10.60^
	*R*^2^ = 0.9998	*R*^2^ = 0.9961	*R*^2^ = 0.9999

## 5 Conclusion

In this study, we connected an elastomer, a viscosity element, a Kelvin element, and a viscoelastic–plastic element in series to construct a nonlinear viscoelastic–plastic creep damage model. The material parameters and damage factor were also introduced into the model. One- and three-dimensional creep equations were derived, and triaxial creep data were used to determine the model parameters and to validate the model. The major findings are summarized below:

1. A nonlinear viscoelastic–plastic creep damage model that effectively models the nonlinear creep characteristics of rocks under freeze–thaw cycles was constructed by connecting an elastomer, a viscoelastic element, a Kelvin element, and a viscoelastic–plastic element in series.
2. The established model better reflects the creep behavior of rock in the attenuation creep stage, constant creep stage, and accelerated creep stage. The quantitative relationship between the parameters and the number of freeze–thaw cycles was obtained for the third stage.
3. The nonlinear viscoelastic–plastic creep damage model is simple and reliable, and the parameters are few and easy to control. The model curve and test data have a good fit, with a fitting correlation coefficient close to 1, indicating that the model effectively describes the creep characteristics of rock under different freeze–thaw cycles and verifying the rationality of the model.
4. The quantitative relationship between model parameters and freeze–thaw cycles can be characterized as follows: with the increase in the number of freeze–thaw cycles, parameters *G*_1_, *G*_2_, and *η*_20_’ decrease exponentially, whereas parameter *λ* increases exponentially. The effect of freeze–thaw times on parameter *η*_1_’ can be expressed by a cubic polynomial: *y = ax*^*3*^ *+ bx*^*2*^ *+ cx + d*.
